# Cognitive Processes Unfold in a Social Context: A Review and Extension of Social Baseline Theory

**DOI:** 10.3389/fpsyg.2020.00378

**Published:** 2020-03-10

**Authors:** Elizabeth B. Gross, Sara E. Medina-DeVilliers

**Affiliations:** ^1^Department of Psychology, Randolph College, Lynchburg, VA, United States; ^2^Department of Psychology, University of Virginia, Charlottesville, VA, United States

**Keywords:** social baseline theory, Bayesian theory, economy of action, attachment theory, dyadic interactions

## Abstract

Psychologists often assume that social and cognitive processes operate independently, an assumption that prompts research into how social context *influences* cognitive processes. We propose that social and cognitive processes are not necessarily separate, and that social context is innate to resource dependent cognitive processes. We review the research supporting social baseline theory, which argues that our default state in physiological, cognitive, and neural processing is to incorporate the relative costs and benefits of acting in our social environment. The review extends social baseline theory by applying social baseline theory to basic cognitive processes such as vision, memory, and attention, incorporating individual differences into the theory, reviewing environmental influences on social baselines, and exploring the dynamic effects of social interactions. The theoretical and methodological implications of social baseline theory are discussed, and future research endeavors into social cognition should consider that cognitive processes are situated *within* our social environments.

## Introduction

With the start of the cognitive revolution in the mid-20th century came a renewed interest in applying the scientific method to studying the mind. Simultaneous advances in technology and computer processing strongly influenced psychologists’ approach to their endeavors. Scientists of the time applied the current technological terminology and definitions to the mind. The mind was comprised of cognitive *processes* that operated on *representations*, and often in *serial* manner. A particularly tricky aspect of this approach was isolating mental processes. To do so, the researcher must tightly control for any and all confounding variables, isolating individuals, and specifying appropriate control conditions, to ensure they were, in fact, measuring the variable of interest. The ingenuity and creativity of early cognitive scientists are impressive, and there is no doubt their efforts resulted in psychological advances too numerous to quantify.

Recently, researchers have embarked on studies in embodied and social cognition, whose primary area of interest is to move beyond isolated cognitive process and study instead how our physiological and social environments interact with our cognitions, respectively. Still, these endeavors in social cognition often still function from an isolationist perspective. For embodied cognitive psychologists, the focus still revolves around how the *individual*’s physiology affects cognition. For social cognitive psychologists, the predominant assumptions are that social effects on cognition are either the results of an individual’s top-down processes or involve separate cognitive processed devoted specifically to social situations.

Conversely, researchers have proposed social baseline theory, which suggests that the default in cognitive processes is to assume the availability of social resources (Beckes and Coan, 2011; Coan and Maresh, 2014; Coan and Sbarra, 2015). If social baseline theory is correct, then it is not a question of which cognitive processes are involved in social situations. Rather, it is more appropriate to state that our physiological, neural, and cognitive processes are almost always situated within social situations. However, the effect of social environments is more complicated than simply a net positive/negative effect. Indeed, there are individual and environmental differences as well as group dynamics that must be taken into account. In the current paper, we will review the empirical evidence supporting social baseline theory, extend the theory to basic cognitive processes, highlighting research on individual differences, environment effects, and dyadic interactions, and offer suggestions for future research to move the field forward in studying the interaction between cognitive processes and social environments.

## Review of Social Baseline Theory: What Is a Baseline

Social baseline theory first rests on the assumption that individuals operate under an economy of action. That is, all organisms must take in more energy than they expend (Proffitt, 2006; Beckes and Coan, 2011). This requires that individuals maintain homeostasis around a baseline. We outline two physiological examples of a baseline, blood glucose, and thermoregulation, as we will later summarize published research supporting social baseline theory involving these physiological processes.

Glucose is a necessary component of human functioning that operates via a feedback system. When sugar is consumed, the body keeps a baseline level of glucose in the bloodstream, ready for use. If an excess of glucose is present, it is stored as glycogen in the liver, and when the amount of blood glucose drops below the baseline, glycogen is released from the liver into the bloodstream (Benton et al., 1996). Thermoregulation is yet another physiological process that also maintains homeostasis around a baseline via a feedback loop. The average temperature set point for humans is 98.6 degrees Fahrenheit. When the preoptic area/anterior hypothalamus (POA/AH) receive messages regarding changes in temperature from thermoreceptors in the skin, a cascade of hormonal responses trigger physiological changes to increase or decrease our core temperature as needed (Satinoff and Rutstein, 1970; Van Zoeren and Stricker, 1977). For example, if a body’s internal temperature drops below the baseline, individuals shiver to produce body heat, and then produce thyroid hormone to raise overall metabolic activity, which subsequently raises body temperature (Barnes et al., 1976). For both blood glucose and thermoregulation, fluctuations around the baseline are met with compensatory actions to maintain homeostasis. Similar to our body’s physiological processes, social baseline theory proposes that our neural and cognitive processes also operate around a baseline.

### Social Baseline Theory and Evidence

Social baseline theory asserts that the baseline for neural and cognitive processes is to assume close proximity to social resources. It is not the case that the presence of other individuals brings us above our baseline and adds cognitive processes to represent added social resources, but rather, social resources put individuals at their baseline. To study the neural and cognitive processes of an individual alone is to study them below their baseline. In an economy of action framework, when individuals are meeting their social baseline, they will expend minimal cognitive effort. However, when individuals are alone, or below their social baseline, you would expect to see additional neural, cognitive, and behavioral processes to compensate for the deficit. In other words, individuals will spend more cognitive effort and energy when they are alone rather than when they are situated in their baseline social network.

An illustration of this principle can be found in taking a Bayesian perspective of cognitive processes. In a Bayesian approach to decision-making, individuals calculate the costs and benefits of an action based on previous knowledge and experience, or priors. As individuals acquire new experiences and situations, the priors are updated (Anderson, 1998). If the social environment is meeting an individual’s baseline expectations, there is no need to expend energy or use cognitive processes to update the priors. However, if individuals are below their baseline, specifically, alone or without social support, they will expect to expend more energy in updating priors to calculate the least costly decisions or actions. There is, in fact, physiological and neural evidence to support this social baseline approach, which we review in turn.

In thermoregulation, the physiological responses to regulate temperature, as described above, function through a feedback mechanism. Behaviorally, individuals can also function to maintain core temperature prospectively (Stearns and Stephen, 1992; IJzerman et al., 2015). When it is winter, individuals do not wait to step outside and shiver before acting, but rather put on coats, gloves, and scarves before leaving the house. Since raising body temperature is metabolically expensive, this predictive response is bioenergetically less costly.

Similarly, social environments can reduce the cost of thermoregulation. There is evidence that animals will also behave prospectively to thermoregulate by utilizing their social environments, and this action is metabolically efficient. For example, in cold temperatures, Chilean rats’ metabolic rate is reduced by almost half when huddling in groups of three or five compared to an alone condition (Nunez-Villegas et al., 2014; IJzerman et al., 2015). In this case, animals utilize their social environments to efficiently regulate even the most basic of physiological processes, thermoregulation, to protect homeostasis around a baseline.

Moreover, there is evidence that suggests humans incorporate expectations about their social networks in order to efficiently thermoregulate. In one study, researchers continuously measured participants’ peripheral body temperature while they were socially excluded or included. Participants’ finger temperatures dropped relative to baseline during social exclusion but increased relative to baseline during social inclusion (IJzerman et al., 2012). These results could be due to the stressful nature of social exclusion and the positive nature of social inclusion, as previous research shows that stress results in peripheral vasoconstriction, while positive affect results in peripheral vasodilation (Rimm-Kaufman and Kagan, 1996). Social baseline theory also provides a framework for understanding these results. Cutaneous vasoconstriction and vasodilations are part of the homeostatic process of thermoregulation. Peripheral vasoconstriction reflexively serves as a defense mechanism to conserve internal body temperature, and vice versa for peripheral vasodilation (Alba et al., 2019). In social baseline theory, social inclusion indicates a positive social environment, and the presence of added positive social support would have pushed individuals above their baseline social expectations. Presumably, a positive social support condition might also signal to individuals that less conservation of resources is required, leading to vasodilation and a rise in finger temperature. On the other hand, the negative social support condition would have signaled added costs to acting in the environment and fewer available resources, resulting in vasoconstriction and a lower finger temperature.

Social networks may also influence behavioral decisions regarding another physiological resource, glucose consumption. In a correlational study, individuals that reported more social isolation also reported consuming more sugary beverages on average (Henriksen et al., 2014). This effect remained even after controlling for physiological factors such as weight and mood. Again, if our cognitive baseline is to expect a social environment, isolated individuals that fall below that baseline would need to stockpile physiological resources to compensate for expected costs of acting.

Finally, there is neural evidence to support social baseline theory. In a seminal study, researchers used fMRI to measure neural activity in participants expecting a mildly painful electric shock (Coan et al., 2006). They found that neural circuits typically associated with emotion regulation, for example, the dorsolateral prefrontal cortex (dlPFC), were *less* active when social support was provided. This finding was recently replicated; participants under threat of electric shock showed significantly less neural activity in both the dlPFC and dorsal anterior cingulate cortex (dACC) when holding hands with a partner relative to an alone condition (Coan et al., 2017).

Furthermore, these findings have been extended to actual pain experiences, rather than just threat-related neural activity. Holding hands with a partner reduced activity in a pain-related neural circuit, and this reduction in activity mediated self-reported pain intensity and unpleasantness (López-Solà et al., 2019). These results are initially surprising. Typically, adding extra cognitive processes during neuroimaging should increase the activity in involved neural circuits. Instead, introducing the presence of a socially supportive environment *decreased* activity in associated neural networks. Again, such results suggest that the baseline neural and cognitive states are to assume social support. When an individual is deprived of social support, as in the alone condition in the above hand holding studies, then extra cognitive processes are required, rather than the reverse.

In sum, both physiological and neural studies provide initial support for social baseline theory. Thermoregulation studies indicate that both animals and humans will behave prospectively to conserve physiological resources around a baseline, and that social environments are incorporated into an economy of action framework regarding said physiological resources. More importantly, there is strong neural evidence that participants’ baselines are, in fact, social. This necessarily means that to study individuals while alone is to study them with extra costs to functioning in the environment, and therefore added cognitive and neural processes to a baseline.

## Extensions of Social Baseline Theory

Social baseline theory can be extended beyond physiological processes and neural mechanisms of behavior to explain previous findings in basic cognitive processes, such as visual perception, memory, and joint attention, reviewed in turn below. This brief review is not intended to be comprehensive, but to illustrate how social baseline theory can integrate and predict social influences in basic cognitive tasks. More importantly, social baseline theory should be expanded to include a discussion on individual differences. It does not necessarily follow that all individuals have the same social baselines nor that all social influences are necessarily positive. As such, we highlight research suggesting that early life experiences can set individual social baselines, and discuss how social interactions can cause transient fluctuations in individual baselines. Finally, these considerations still focus entirely on the effect of social baseline theory at an individual level. We propose that future research in both social baseline theory and social cognition should consider methodologies that incorporate and measure dynamic social interactions.

### Social Baseline Theory in Cognitive Processes

In visual perception, individuals overestimate the slant of hills. For example, on average, a 25° hill is reported to appear 45° (Proffitt et al., 1995; Schnall et al., 2008). Anecdotally, this phenomenon is illustrated by the famous Lombard Street in San Francisco, which appears incredibly steep but measures, in fact, around 18°. Additionally, there is a growing body of evidence that suggests that both hill and distance perception are sensitive to physiological resources. Hills appear steeper and distances farther away when individuals are less physically fit, elderly, fatigues, and have lower blood sugar (Proffitt et al., 1995; Proffitt, 2006; Schnall et al., 2010). In fact, measures of individual differences in physical fitness will predict distance estimates before any interventions in a lab; individuals who are more physically fit will perceive objects to be closer than those who are less physically fit (Zadra et al., 2010). This evidence strongly suggests that changes in conscious visual experiences are due to changes in physiological resources. In other words, the visual system is sensitive to a body’s ability to act in the world, which is reflected in the conscious visual experience of the environment around us.

As previously discussed, social baseline theory asserts that social resources will serve as a signal that cognitive and physiological loads are lower than when acting alone. Therefore, in terms of visual perception, social baseline theory would predict that the presence of supportive social resources would result in a smaller overestimation of hill slant. Alternatively, the presence of negative social resources will increase cognitive load, and would increase the slant of the hill. This prediction is supported by research. Schnall et al. (2008) found that when individuals imagined supportive others, they reported that hill slants appeared less steep than those who imagined a negative individuals. It seems that even within basic cognitive processes, such as visual perception, our minds are not only sensitive to cognitive loads and the cost of acting in the environment, but they also incorporate our social environments into these cost and benefit calculations.

Social effects have also been documented in another basic cognitive process, memory. In memory recognition tasks, social groups outperform individuals (Clark et al., 2000; Rossi-Arnaud et al., 2011). This is expected, and the results fit within a social baseline framework. The presence of others in a memory task allows a distribution of the cognitive load among individuals, which would result in improved performance. In collaborative memory tasks, individuals will typically encode items separately, and recall items either alone or in collaborative groups. Unsurprisingly, collaborative group recall surpasses individual recall (Andersson and Rönnberg, 1996; Weldon and Bellinger, 1997). However, group recall often will fall short of pooled individual efforts; that is, the sum of separate individual efforts at recall will surpass the average recall of the same individuals in a group (Andersson and Rönnberg, 1996; Weldon and Bellinger, 1997). On the surface, these findings appear contrary to social baseline theory. Still, further investigations into collaborative memory tasks reveals that the decline in group recall is because groups likely create less successful cues during encoding and inhibit successful memory retrieval strategies (Basden et al., 1997; Finlay et al., 2000; Barber et al., 2010; Rajaram, 2011). In fact, when investigating friend versus non-friend pairs, the decline in collaborative group recall was less pronounced for friend groups versus non-friend groups (Andersson and Rönnberg, 1996). Social effects on cognitive processes are not always positive, but these findings highlight the importance of investigating cognitive processes from a social baseline perspective. To quote previous researchers, “Humans routinely encode and retrieve experiences in interactive, collaborative contexts. Yet much of what we know as researchers comes from research on individuals working in isolation” (Barber et al., 2010).

Finally, literature on joint action provides a particularly strong case and example of social baseline theory. In joint action, individuals are required to coordinate their actions to achieve a common goal, which necessarily includes sharing representations on the environment, and predicting their own and others’ actions (Sebanz et al., 2006). Humans have evolved to function optimally in our ecological niche, and as social animals the cognitive mechanisms for joint action would have evolved to coordinate behaviors in a social environment (Marsh et al., 2009). van Schie et al. (2004) found that when monitoring others’ performances in task sharing, the same neural mechanisms were activated as if the individuals were performing the action themselves, with errors in others’ actions resulting in increased neural processing. Furthermore, evidence suggests that individuals may automatically represent others’ intended action goals. For example, reaction times in “go-nogo” tasks were significantly slower in the presence of others, even when individual participants were responding to different stimulus-response instructions and had no visual information regarding others’ actions (Sebanz et al., 2003, 2005). In other words, even in tasks not requiring collaborative actions and even when others’ actions were not visible, individuals still were representing others’ actions in the social environment.

### Individual Differences in Baselines

While research supports that individuals’ baselines are on average social in nature, the theory does not claim that all social baselines are identical. Indeed, one must consider that there are individual differences in social baselines. Once again, a Bayesian perspective is useful when considering individual baselines. When relying on others in the face of a threat, humans trust that they are operating in a social environment that provides support. However, this is risky, because if our relational partners are not in fact engaging in some amount of vigilance on our behalf, then individuals place themselves at increased risk by relaxing our own vigilance processing. So how do people know who to trust? According to Bayesian theory, our brain places “bets” on the reliability of a social resource based on a prior probability distribution of past social experiences, and the deployment of personal resources are in turn based on this prediction. In this way, one’s history of relationships may account for individual differences by influencing priors. Early familial support and attachment (Coan et al., 2013) and social capital (Lee, 2013; Liu et al., 2013) could be viewed as sources of useful information for these priors. Subsequently, maternal attachment and social capital have interactive effects on physiological behaviors, epigenetics, and neural responses to threat. In this section, we discuss how information from our social environment helps form our priors that in return produces an individual and unique social baseline that alters responses to the environment.

Experiences in early childhood with caregivers form our attachment styles, which in turn form the basis through which individuals approach later relationships (Bowlby, 1969). Children who experience warm, supportive caregivers responsive to their needs develop a secure attachment style, whereas children whose caregivers do not meet their needs will develop an insecure attachment style (Ainsworth, 1978; Bartholomew and Horowitz, 1991). These early life experiences shape expectations about future relationships. From a Bayesian perspective, they set our priors such that secure individuals expect others to be reliable and supportive, and vice versa for insecure individuals, which has been demonstrated in research. For example, individuals with secure attachment styles are more likely to seek social support and perceive provided support as positive, whereas insecurely attached individuals do not (Collins and Feeney, 2004). Like attachment styles, we argue that early childhood experiences and the larger environments within which individuals are situated can shape our social baselines such that each individual has their own unique social baseline.

Recently published research in thermoregulation provides support for individual differences in social baselines. In a pre-registered, replicated study (IJzerman et al., 2018), participants held either a warm or cold cup, ostensibly to rate it on a consumer survey, recalled the first five people that came to mind, and finally rated how close they felt to each person. Participants also answered an attachment style questionnaire [Experiences in Close Relationships (ECR)], from which researchers derived a set of items to measure individual differences in positive and negative relationship experiences. In the cold condition, individuals that reported positive relationship experiences were more likely to recall closer others, and vice versa for the warm condition. This is consistent with previously discussed research in social thermoregulation (Fay and Maner, 2012; IJzerman et al., 2013). However, individuals that reported negative relationship experiences showed the opposite effect. Those individuals were *less* likely to recall close others in the cold condition, and vice versa for the warm condition. These effects are viewed as compensatory effects (IJzerman et al., 2018), and can be explained by individual differences in our social baselines. For individuals with positive relationship experiences, their priors are such that others represent a reliable source of social support. In other words, they have a higher social baseline and so they are more likely to recall closer individuals. On the other hand, individuals with negative relationship experiences will not expect others to be a reliable source of social support or warmth, and so others represent an extra added cost to functioning in the environment. In the cold condition, which presumably invoked the potential for a metabolically costly physiological response, participants with a lower social baseline were less likely to think of close others because of this potential cost. This highlights the importance of investigating individual differences in social baselines. Without measuring previous experiences in relationships, and considering individual differences in these priors, it is likely the social thermoregulation effect would not have been replicated.

Individual differences in personality traits related to interacting with social environments will also produce varying individual social baselines. One such example is extroversion; Esyenck’s biologically based theory of extroversion suggests that extroverts typically seek out interactions in social environments because they have a lower physiological arousal baseline than introverts (Matthews and Gilliland, 1999). In socializing and interacting with others, extroverts are energized, thereby raising their arousal baselines. Introverts, on the other hand, have higher arousal baselines and so at times prefer to withdrawing from social stimulation. Much as in the research of physiological thermoregulation, differences in extroverts and introverts come from individual’s utilizing the social environment to regulate physiological arousal. This theory is supported by physiological evidence. Results in EEG studies show that extroverts have lower baseline cognitive activity levels than introverts (Beauducel et al., 2006; Hagemann et al., 2009). Differences in arousal levels between extroverts and introverts also have behavioral implications. For example, extroverts are less successful in vigilance tasks, which benefit from higher levels of arousal (Beauducel et al., 2006; Cox-Fuenzalida et al., 2006). Interestingly, these effects are predicted by social baseline theory. Social groups allow individuals to offload visual tasks to the group. Extroverts, when tested alone, are below their social baseline and unable to offload the cognitive load of the task. As such, their social baseline is not met, their physiological arousal levels are lower than baseline, and they perform worse on vigilance tasks. Conversely, when introverts’ are tested alone, they are closer to their social and arousal baselines, and so their performance in the vigilance task does not suffer compared to extroverts.

Additionally, personality traits, such as extroversion, also alter individuals’ responses within social environments. Extroverts not only report larger social networks, but they also are more likely to seek social support resources and perceive more available social support in their networks (Swickert et al., 2002). In other words, there are individual differences that mediate a response to social support. For example, there are gender-specific differences following a social exclusion task (Seidel et al., 2013), and those higher in trait anxiety exhibit significant differences in self-report measures and neural response following social exclusion (Heeren et al., 2017). Even more ephemeral changes in an individual’s behavior, such as physical perspective and cognitive stance, are associated with perceptual differences of another’s pain and pleasure (Fusaro et al., 2019). Individual differences not only set different social baselines, but these differences also alter how we respond to provisions of social support and social processes, such as social inclusion and exclusion.

Evidence of individual baselines is present in neural research as well. Similar to physiological measurements of individual baselines, neural activity is dependent on one’s social environment. Enormous individual differences exist in coping with environmental stressors and creating and maintaining relationships with others. In a moment of threat, these differences include how one perceives and interprets a situation; one may perceive a loud crash during the night as someone breaking into their house, or as their cat knocking over a lamp, thus interpreting the sound as threatening or just annoying. This difference in interpretation leads to significant differences in cascading biological and neural reactions.

For instance, social environments characterized by supportive relationships regulate hypothalamic-pituitary activity, such that higher self-ratings of general health correspond with decreased hypothalamic activity during supportive hand holding in a threat task (Brown et al., 2017). Therefore, associations between an individual’s social support and health outcomes are partly mediated through the social regulation of hypothalamic sensitivity to threat. This may indicate that hypothalamic sensitivity to threat depends on the individual and the individual’s response to social support. And thus, how an individual responds to social support in the face of threat has downstream health outcomes.

Furthermore, the environment in which one develops influences an individual’s social baseline. In one study, Gonzalez et al. (2017) used a validated measure of life history that quantified the relative harshness and instability experienced during an individual’s development. They then investigated the interaction between life history, neural activity during negative stimuli, and oxytocin receptor gene (OXTR) polymorphisms on mental health outcomes. Findings suggest that economic privilege and specific types of epigenetic variability may calibrate social motivational neural systems for better or worse. For individuals with epigenetic predispositions that decrease the expression of certain oxytocin receptors, a stressful environment during critical periods of development interacts with these predispositions such that those individuals are more likely to develop anxiety and depression. In other words, the environment, both social and otherwise, that characterizes an individual’s development has a significant effect on one’s anxiety and depression dependent on the additional individual variability of one’s genes. Environmental demands during early development can have ontological phenotypic effects that culminate in subsequent mental and physical effects.

We argue that personality, attachment style, personality, and life history, including genetic predispositions, are important latent variables that compose an individual’s traits, but it is not a comprehensive list of individual differences. Rather, any early life experiences and individual traits that alter expectations regarding the reliability and usefulness of social resources in the environment will produce a unique social baseline for each individual. Because of varying social baselines, the same social environment will produce differential effects on physiology, cognition, behavior, and neural mechanisms. Furthermore, we expect these individual differences in social baselines to be lasting, akin to a biological set point, but certainly do not claim social baselines are permanently fixed.

### Environment’s Effect on the Individual

**FIGURE 1:**
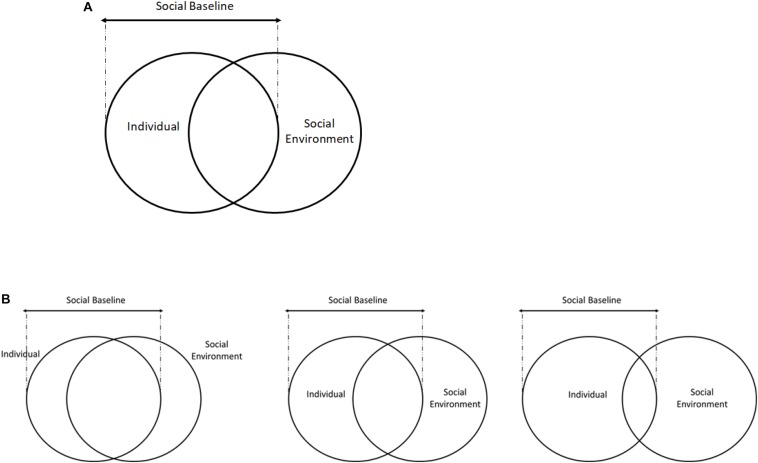
The individual and social environment overlap represent social baselines. Early life experiences and personality traits are part of the individual differences that constrain social baselines to a range **(A)**, while social environments and dynamic interactions will cause temporary fluctuations in baselines around the set point **(B)**. In **B**, the middle represents the social baseline set point as determined by early life experiences and personality traits. The left circle indicates a transient increase in the incorporation of the social environment and social resources into the self due to changes in the current social environment, while the right circle demonstrates the opposite.

While individuals have a semi-permanent set point for their social baselines, the immediate environment can cause fluctuations around these set points. Early environments affect individuals by influencing and updating priors, turning gene expression on and off, and ultimately determining one’s baseline. Aside from individual differences, the immediate context of social relationships also has a powerful transient influence on our cognitive and neural processing and can temporarily alter the set point of an individual’s social baseline (see Figure 1). Importantly, social affiliates are often part of the immediate environment, and one’s relationship with individuals and groups determine the quality of social resources one receives. It is important to note that social resources are not always positive; social environments, while mostly beneficial, might also incur a cost. Furthermore, social environments are not static, but necessitate dynamic responses to others.

Both threat and attachment figures are critical parts of the environment influencing how one then allocates cognitive resources, for better or worse. In the case of a *strong, positive* attachment figure, social relationships (i.e., social resources) buffer environmental threats, likely by changing how individuals perceive the threats. A trusted and interdependent conspecific can provide help in identifying and acquiring resources (e.g., food, shelter), vigilance for environmental threats, and help in caring for offspring, to name a few. These conspecifics *share* in the work for personal and genetic survival. This shared problem-solving, also known as load sharing (Coan, 2008), is a process by which individuals distribute effort in responding to environmental demands. In contrast to risk distribution, which mainly relies on an optimal number of conspecifics, load sharing relies on the relationship between said conspecifics. By sharing a goal with trusted conspecifics, the perceived energy required to achieve that said goal is also shared. Animals share in caring for young (Ehrenberg et al., 2001), acquire food together (O’Brien et al., 2005), and contribute to being vigilant for enemies (Davis, 2010). However, there must be a foundation of shared goals, such as a desire to perpetuate one’s own genes, in order to motivate animals and humans alike to work together and share resources. This makes social relationship economically beneficial because they help achieve goals with shared cognitive resources.

We argue that social relationships, alongside load sharing, create a unique interaction and utilization of one’s social environment. These differences have been investigated, namely, in two ways: (1) by observing individual responses to a stimulus and (2) how two or more individuals react simultaneously and dynamically with each other to the same stimuli. We discuss how the interplay between an individual and his or her social environment can have positive and negative impacts for the individual and the overall social relationship.

Social *environments* are malleable to the extent that social *relationships* are malleable. By changing how one individual perceives and interacts with a partner, positive and negative effects of threat perception and allocation of resources may change as well that constitute, in part, social baselines. In this respect, by studying an individual in isolation (as opposed to dyadic measurements), we can isolate and specify individual variables that may contribute to overall health and well-being. Johnson et al. (2013) introduced an empirically supported therapy strongly focused on repairing adult attachment bonds to distressed romantic partners. They observed a significant decreases in the neural activation and downstream regulation of neural threat response post-intervention when holding the hand of their romantic partner, particularly in brain regions associated with moderating negative affect (Etkin et al., 2011) and supporting cognitive reappraisal (Ochsner and Gross, 2005), such as the dACC and prefrontal cortex (Johnson et al., 2013). Additionally, prior research suggests that PFC-mediated work is computationally, biologically, and neurally costly (Halford et al., 1998; Dietrich and Horvath, 2009); therefore, a decrease in PFC-activity post-intervention also suggests conservation in cognitive resources. By improving the bond and interdependence between participant and romantic partner, social regulatory processes also improved by changing the way the brain encodes and responds to threats, likely harnessing more social and cognitive resources provided by the romantic partner and maximizing the benefits of load sharing. In other words, improvement in relationship quality with relational partners resulted in a higher social baseline, which translated into decreased threat perception when in proximity to the relational partner.

Social relationships can also have negative effects on threat perception and resource allocation. Co-rumination is a repetitive and cyclical discussion of a problem between two or more interdependent people without an objective to solve said problem. This process may heighten threat perception and response (Parkinson and Simons, 2012) and is associated with an increase in emotional distress (Calmes and Roberts, 2008; Smith and Rose, 2011). Thus, it might require more resources on the part of both partners in a dyad to combat stressors, which would lower each individual’s social baseline. As a result, each individual in the dyad group would not pool social resources, and as such, each individual has a heightened threat response to stressors in the environment. However, these studies still investigate effects from an individual’s perspective. They help us isolate particular psychological mechanisms of functioning but miss the dynamic interaction of social groups. Understanding this dynamic interaction is important because we can make psychological inferences from physiological and neural influences—the extent to which one dyad member’s physiology or neural underpinnings predicts the other dyad member’s physiology at a future time point (Thorson et al., 2017). This importance is compounded further by our assertion that an individual’s cognitions are influenced within the social context.

In sum, we see individualized baselines in multiple domains: blood cortisol levels, thermoregulation, hypothalamic activity, and neural reactivity. Our baselines determine the physiological and cognitive responses we observe. While humans, in general, assume social support, there are individual differences in how one seeks out, receives and gives social support, and generally utilizes social resources. The following section moves away from the individual and describes how specific social environments may impact these individual differences and how in return, our individual baselines may determine how we respond to our environment.

### Dynamic Extension of Social Baseline Theory

The above research is an example of environments’ effects *on an individual.* However, we also argue that individual and social environments are not completely separate entities, but instead are co-existing and reciprocal systems that produce downstream reactions throughout the social context, not just on one individual. For example, research on the relationship between the self and another conspecific indicates that the concept of the self often incorporates others (Galinsky et al., 2005; Beckes et al., 2013). Self-expansion theory posits that the more familiar we are with a particular person, the more we perceive that person as ourselves (Aron and Aron, 1996). This concept of the “self-other overlap” extends to neural systems. Self-focused neural threat activity is robustly correlated with friend-focused neural threat activity but not stranger-focused neural threat activity (Beckes et al., 2013). Increasing levels of overlap between neural representations of self and other suggest that an individual may not be completely separate from more familiar conspecifics. Additionally, enfacement theory posits that synchronous stimulation and movement of self and other create a subjective illusion in which the other appears as the self (Porciello et al., 2018). Participants exposed to synchronous stimulation showed more merging of self and the other than participants exposed to asynchronous stimulation (Paladino et al., 2010). This multisensory integration can affect social perception and create a sense of self-other similarity or discrepancy. Recent research on the enfacement effect also provides evidence that a social environment can evoke changes in self-identification (Paladino et al., 2010; Tajadura-Jiménez-Jiménez et al., 2012; Porciello et al., 2018). The incorporation of stimuli in the social environment into the concept of the self extends to an individual’s social baseline (see Figure 1).

The social environment contains a multitude of threatening situations that produce a physiological stress response. By measuring joint and dyadic responses between two conspecifics, we can also investigate how a dyad may actually coregulate, as opposed to investigating how any one individual regulates responses to the environment. This coregulation, or synchrony, may reflect a homeostatic, regulatory process in which interdependent dyads, such as romantic partners, jointly pull each other toward a baseline level characterized by greater stability in the system.

Research on the physiological synchrony and dynamics between dyads suggest that the interplay between a dyad’s physiological responses is associated with positive and negative individual and interpersonal functioning outcomes (Pauley et al., 2015; West et al., 2017; Mckillop and Connell, 2018). Linkage in multiple systems was positively associated with indices of relationship connectedness, such as the amount of time spent together and the ability to identify the emotions of one’s partner (Timmons et al., 2015). However, synchrony in cortisol levels of marital partners is negatively associated with relationship satisfaction (Timmons et al., 2015). Additionally, mothers’ stressful experiences are considered “contagious” to their infants, and members of close pairs, like mothers and infants, can reciprocally influence each other’s dynamic physiological reactivity (Waters et al., 2014). Dyadic interactions may also highlight more complex associations between one’s social environment and individual outcomes. For example, marital satisfaction may buffer spouses from their partners’ negative mood or stress state (Saxbe and Repetti, 2010). Physiological linkage may confer benefits but also may put couples at risk if they become entrenched in patterns of conflict or stress. Overall, this evidence suggests that any effects should be considered in light of dynamic responses among one’s social environment, particularly between dyads.

Emerging research of social networks offers a valuable extension of dyadic processes and a more thorough understanding of the relationship between an individual and not only their assumed social environment, but also within an ecologically valid context. Recent work by Morelli et al. (2018) highlights the importance of studying not just the individual within the social environment but also the social environment as a whole. Participants identified different types of relationships with members of their proximate social environment and completed self-report measures of personality. A dynamic social network was created based on these measurements. By examining individuals within a social context, they found that those high in well-being (i.e., life satisfaction and positive emotion) were central to networks characterized by fun, whereas individuals high in empathy were central to networks characterized by trust (Morelli et al., 2018). This provides evidence that well-being is socially attractive, whereas empathy supports close relationships. We posit that individuals who have higher quality social relationships with multiple conspecifics in their environment have more social resources and are, therefore, more likely to have a higher social baseline. Furthermore, this emphasizes the importance of studying psychological constructs within a social context.

## Summary and Conclusion

Social baseline theory suggests that, as a social species, our baseline assumptions in physiological, cognitive, and neuropsychological processes are situated in social contexts. In other words, in an economy of action framework our baseline defaults to expect social resources and social support. As such, it may be the case that there are not separate cognitive processes devoted specifically to the social environment, but cognitive processes may be automatically situated in social environments. We have reviewed evidence that suggests that our social baselines have surprising influences on our physiological, neural, and cognitive processes. These results suggest that cognitive processes are *generally* situated within our social baseline and that even in environments that are not inherently social, there may still be social effects.

How far can social baseline theory be extended, and which cognitive and behavioral processes operate outside of social baseline theory? We recognize that social baseline theory may not extend to all cognitive processes. We venture that one example of a cognitive process outside of the influence of social baseline theory is color perception but hesitate to name a list of potential candidates. Social baseline theory hinges on an economy of action framework, which includes conserving physiological and cognitive resources. As such, social baseline theory will extend to cognitive processes to the extent that these processes are resource dependent. However, some cognitive processes would not necessarily rely on physiological and cognitive resources, and so we would not expect social baseline theory to impact such processes.

While being situated in a social environment is the default assumption, social baselines are not ubiquitous for everyone. In fact, there is research that suggests that there are individual differences in the extent to which we are socially situated, and that our social baselines might indicate positive or negative experiences. For individuals with previously positive and supportive social and environmental experiences, their social baseline indicates that others are reliable and will lower their cost of acting in the world. The opposite is true for individuals with previously negative and unsupportive social and environmental experiences. For them, their social baseline indicates that others are unreliable, and so others represent an added cost to acting in the environment. Furthermore, researchers must also consider variability in our social environments, broader environments, and the dynamic responses that occur between an individual and their social environment. Just as individual differences alter the set point of social baselines, previous experiences and overall environments will also create momentary fluctuations in social baselines. From a Bayesian perspective, our priors are not entirely fixed or static, and variability in our social environments or in dynamic responses to individuals will also update our social baselines. Ultimately, investigations into social baseline theory, individual differences, and group and social dynamics are of the utmost importance, as they not only provide support for social baseline theory but might provide a framework for explaining contradictory findings on the effects of social environments.

### Future Theoretical and Methodological Directions

We propose that in order to advance the field, researchers should consider leaving an isolationist approach and embrace an approach that encompasses a theoretical perspective grounded in Bayesian statistics. Because of the aforementioned individual, social, and environmental differences, this suggests that researchers should consider previous experiences that could influence the priors under investigation. This also suggests that certain methodologies could help the field move past an isolationist approach, for example, social network analyses, larger environmental and social contexts, and dyadic and group interactions. This section specifies theoretical and methodological recommendations for future conceptualization and research into cognitive processes that may be influenced by social factors.

A theoretical Bayesian perspective may be useful in conceptualizing the dynamic interaction and temporal nature of a social environment. Our social relationships and environments are not static. So, like physiological mechanisms where individuals have a baseline but fluctuate around that baseline, the reliability of an individual’s relationships and environments *in the moment* will also affect their physiological, behavioral, and neural responses. In environments that are positive and where individuals are trustworthy, this may push us above our social baseline such that individuals will be more likely to offload the cost of acting in the environment, and vice versa for environments and social interactions that are negative. From a Bayesian perspective, this indicates that it is not just individuals’ past experiences that set priors but also our current social environments and interactions—including dyadic interactions with others. That is, our priors will constantly be reinforced or updated given the current situation, which we argue is innately social by context.

Theory grounds sound methodology. We argue that ecological and Bayesian theory provide a solid foundation and framework for understanding human processes. We recommend that these cognitive processes should be studied with social situations in mind in order for a more ecologically valid understanding of human functioning. Additionally, social influences on cognitive processes should be contemplated in a more complex manner that includes the dynamic interplay of group influences. Therefore, we propose the following methodological recommendation for evaluating dyads and groups within a social context.

The first methodological recommendation is to consider both prior and current social context when evaluating outcomes. We argue that past information—including socioeconomic status, prior relationships, attachment history, and life history—are all necessary when understanding current cognitive processes because they determine our social baseline and give context for current measurements. Given that there are many variables to consider, we propose exploratory analyses to investigate whether these variables significantly predict processes or behaviors under investigation. A replication study should then be done to confirm findings.

The second methodological recommendation is to measure social variables in the research setting. This, at a minimum, requires the researcher to consider perceived social support and current social interaction between researcher and participant while moving toward a more ecologically valid design that includes dyads and larger groups. Thorson et al. (2017) provide a guide for considering theoretical and conceptual concerns when designing, implementing, and analyzing dyadic psychophysiological studies. Specifically, different theoretical questions require different physiological measures. For example, researchers interested in co-regulation will want to look at the degree that one partner’s physiology predicts another’s at a following time point (Butler and Randall, 2013; Helm et al., 2014). On the other hand, researchers interested in coupling or synchrony will want to investigate the correlation between two partners’ physiology at the same time point instead (Kinreich et al., 2017). Cacioppo et al. (2007) have provided three dimensions along which psychophysiological relationships can be assessed—generality, specificity, and sensitivity—in order to better understand which physiological response relates to a psychological process. Much of these recommendations center on affective and physiological responses. However, we recommend incorporating these methodological considerations when investigating cognitive processes.

In conclusion, given the evidential support for social baseline theory, we urge researchers to consider that resource-based cognitive processes are generally situated in our social environments, often in surprising and unexpected ways. As such, we suggest a shift away from the assumption that cognitive and social processes are entirely separate, and propose instead that individual differences in our social baselines and the dynamic fluctuations in our social environments inherently shape our cognitive processes. Further research into variations in our social baselines can only serve to deepen our understanding of human behavior and the mind.

## Author Contributions

EG and SM-D conceptualized, drafted, wrote, and edited the article together, with EG taking slightly more lead and responsibility on the project.

## Conflict of Interest

The authors declare that the research was conducted in the absence of any commercial or financial relationships that could be construed as a potential conflict of interest.
